# KINtaro: protein kinase-like database

**DOI:** 10.1186/s13104-024-06713-y

**Published:** 2024-02-16

**Authors:** Bartosz Baranowski, Marianna Krysińska, Marcin Gradowski

**Affiliations:** 1 Laboratory of Plant Pathogenesis, Institute of Biochemistry and Biophysics, Polish Academy of Sciences, Warsaw, Poland; 2 Department of Biochemistry and Microbiology, Warsaw University of Life Sciences (SGGW), Warsaw, Poland

**Keywords:** Protein kinase, Pseudokinase, Phosphotransferase, Structure prediction, HMM, Phosphorylation

## Abstract

**Objective:**

The superfamily of protein kinases features a common Protein Kinase-like (PKL) three-dimensional fold. Proteins with PKL structure can also possess enzymatic activities other than protein phosphorylation, such as AMPylation or glutamylation. PKL proteins play a vital role in the world of living organisms, contributing to the survival of pathogenic bacteria inside host cells, as well as being involved in carcinogenesis and neurological diseases in humans. The superfamily of PKL proteins is constantly growing. Therefore, it is crucial to gather new information about PKL families.

**Results:**

To this end, the KINtaro database (http://bioinfo.sggw.edu.pl/kintaro/) has been created as a resource for collecting and sharing such information. KINtaro combines protein sequence information and additional annotations for more than 70 PKL families, including 32 families not associated with PKL superfamily in established protein domain databases. KINtaro is searchable by keywords and by protein sequence and provides family descriptions, sequences, sequence alignments, HMM models, 3D structure models, experimental structures with PKL domain annotations and sequence logos with catalytic residue annotations.

**Supplementary Information:**

The online version contains supplementary material available at 10.1186/s13104-024-06713-y.

## Introduction

Kinases are among the most crucial enzymes found in all living organisms. They facilitate phosphorylation reactions, transferring phosphate groups from high-energy compounds like ATP to specific target molecules. Within the PKL superfamily, best known are protein kinases responsible for phosphorylating proteins [1]. Additionally, in the PKL superfamily there are small molecule kinases whose substrates include antibiotics and sugars [2], as well as lipid kinases that target membrane lipids like phospholipids and sphingolipids [3–5].

PKL proteins play critical roles in various biological processes, including cell growth, differentiation, and apoptosis. Dysregulation of these proteins can contribute to the development of numerous diseases, including tumorigenesis [6]. Moreover, PKL proteins can act as promoters of antibiotic resistance [2], aid pathogen survival within host cells [5, 7], and serve as effectors influencing cellular processes in affected cells [8]. Consequently, blocking their activity through various types of inhibitors can be crucial in preventing diseases, infections, and treating cancer [9] providing alternative treatments.

Pseudokinases were initially considered to be non-functional relatives of protein kinases that lost their enzymatic activity due to mutations [10, 11]. However, recent studies have revealed that pseudokinases can exhibit alternative enzymatic activities. For example, the coronavirus NiRAN pseudokinase domain transfers nascent RNA to GDP, using an RNA–protein intermediate, and ultimately forming the core RNA cap structure: GpppA-RNA [12]. The SelO pseudokinase performs AMPylation of proteins involved in redox homeostasis [13]. The bacterial pseudokinase effector SidJ polyglutamylates SidE effectors, blocking their activity which consists of phosphoribosyl ubiquitination of host Rab GTPases to evade phagocytosis [14], thus modulating the effect on the host cell. Pseudokinases can also serve as allosteric regulators of protein kinases, influencing their activity [15] or stasis for other proteins (for example as part of the secretion system of bacteria) [16].

A number of databases related to protein kinases are known, e.g., the best known database of human kinases according to Manning’s classification [17] or the database of protein kinases in genomes—KinG [18], which is based on Pfam [19] domains. The Pfam domains are not always well defined in terms of domain boundaries, e.g., the PIP49_C family does not cover the entire PKL fold [20]. The Pan3_PK pseudokinase family lacks the kinase N-lobe [21]. Moreover, the Pfam clan (superfamily) Pkinase does not include all known PKL families e.g., SelO pseudokinase family—involved in redox homeostasis [13] or FAM198 family which has been recently identified as a potential cancer-associated gene [22]. Other examples are Pox_E2-like—a pseudokinase found in Poxviridae [23] or the CLU [24] pseudokinase present in eukaryotes. In addition, a lot of PKL families are not recognized as domains in the Pfam base, for example, the pseudokinase SidJ [14] or the viral pseudokinase NiRAN [12].

The InterPro database, which absorbed Pfam is still missing many known PKL families [25].

Other databases dedicated to protein kinases are specialized, e.g., KLIFS—a database based on structural knowledge allowing to navigate in the space of kinase-ligand interactions [26], KinaseMD—a database collecting most updated information on mutations, unique annotations of drug response, especially drug resistance and functional sites of kinases [27], BYKdb—Bacterial tYrosine-Kinase database [28]. There is no specialized database collecting information on all the proteins that share the common PKL structure.

Earlier, we studied the pan-proteome of the *Legionella* genus bioinformatically. Some of the *Legionella* PKL families seem to be unique to this bacterium [29].

Together with information from our own research, databases and literature our database contains 72 updated and carefully prepared PKL families (Additional file 1: Table S1) and basic information about each family from all domains of life. The available 3D structure models and domain structures can help in search strategies for further PKL homologs [30].

We believe that our semi-automatic approach of constructing the PKL domain family sequence models based on the protein structure model is better than automatic approaches used in other protein domain databases.

The main value of the database lies in its searchable presentation of 32 novel annotated families, previously unrecognized as PKL, along with the assignment of active sites to each family.

## Methods and materials

### KINtaro protein family model

**Fig. 1:**
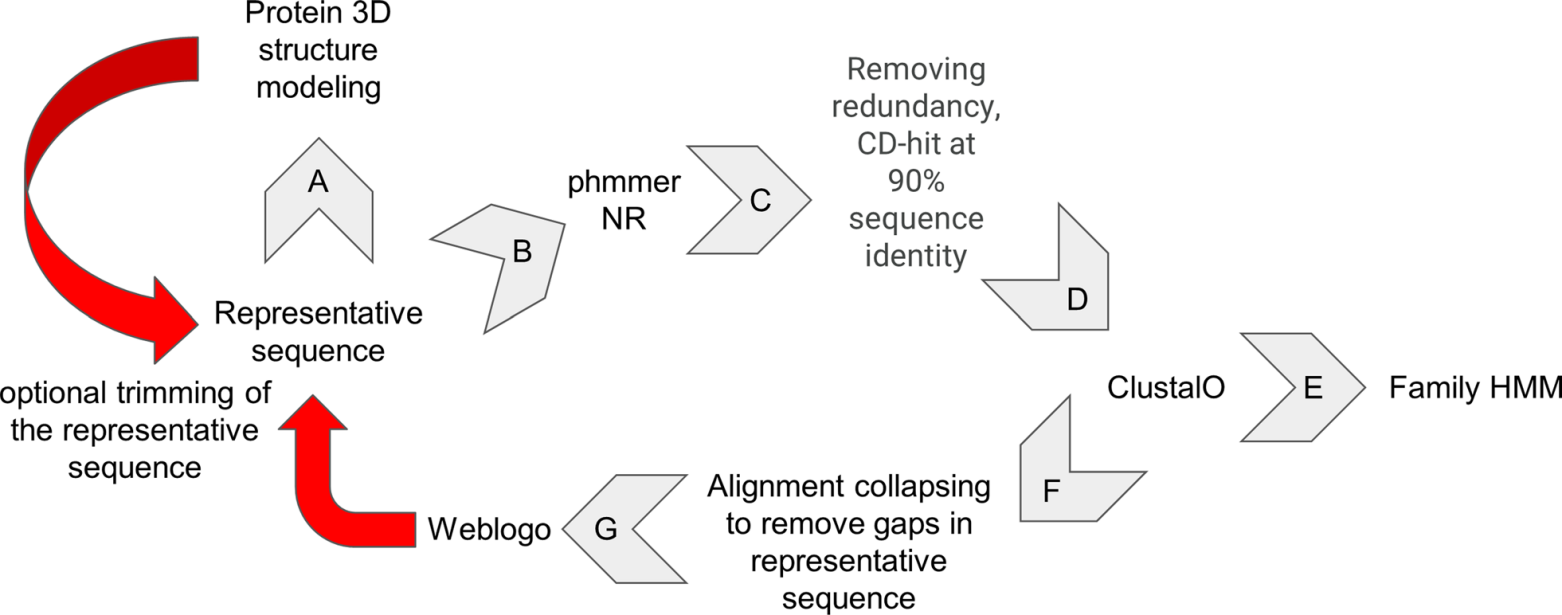
Family model construction scheme

For defining protein kinase families, we adopted an approach similar to the protein database Pfam [19], now part of InterPro [25]. However, Pfam’s “PKinase (CL0016)” protein clan as mentioned before was not adequately updated, and their family models were not always accurate [19]. In our pipeline, we initiated the process of defining a new family with a representative sequence. These sequences were obtained from existing PKL families in Pfam, and also, for families missing in Pfam, from known 3D structures possessing the PKL fold, from novel PKL families described in the literature or from our own sequence/structure searches. Such representative sequences served as a query for 3D structure modeling. Model was created based on the representative sequence (Fig. 1, arrow A) using ColabFold (AlphaFold2 using MMseqs2) or ESMfold, the final model was chosen based on the pLDDT score [31, 32].

**Fig. 2:**
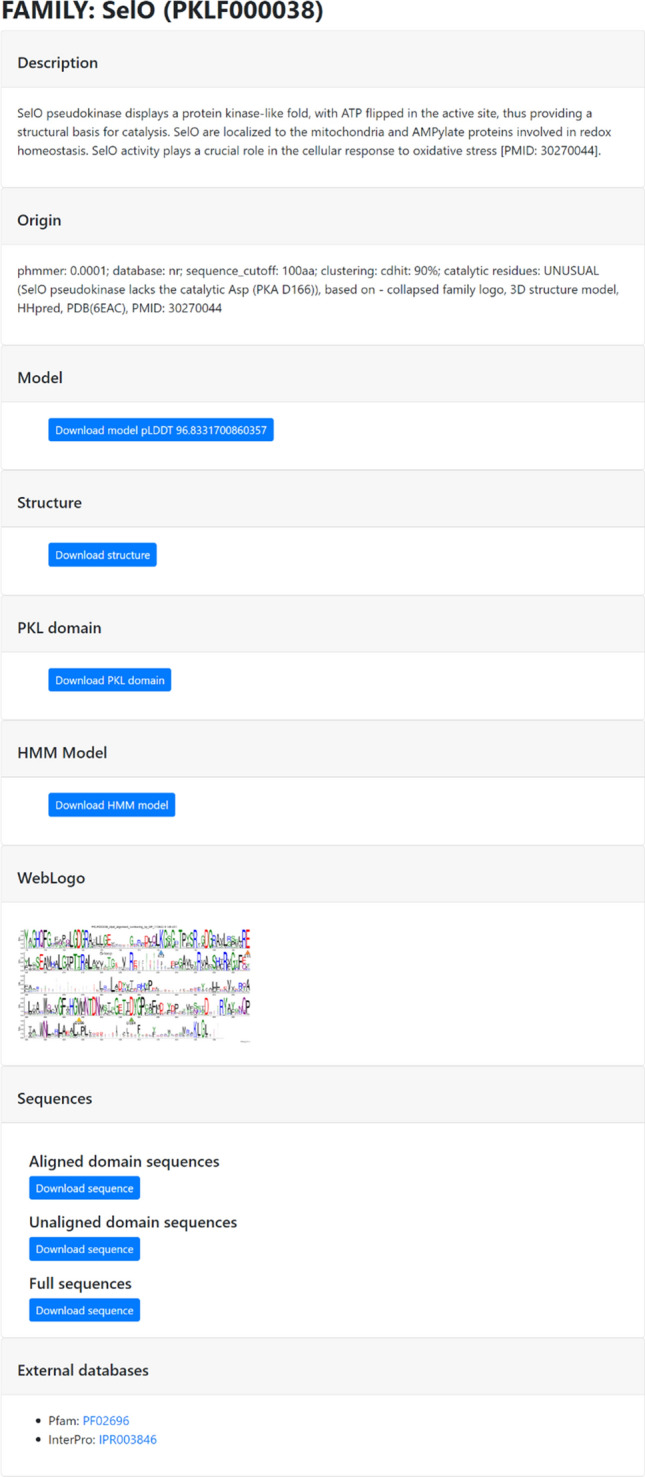
Family card interface

To find all members of a PKL family, a representative sequence also served as a query for phmmer [33] against the NR database [34] with an E-value threshold of 0.0001 (Fig. 1, arrow B). Next, we filtered out homologous sequences shorter than 100 amino acids and clustered them at 90% sequence identity [35] (Fig. 1, arrow C). The clustered sequences were then aligned using the ClustalO program [36] to build the family's hidden Markov model (HMM) [33] (Fig. 1, arrow D and E). The alignment was collapsed, where gaps were removed from the representative sequence (Fig. 1, arrow F). A sequence logo was generated from the collapsed alignment using Weblogo [37] (Fig. 1, arrow G). In the final optional step, an iterative approach was used to enhance the family model by adjusting the domain boundaries, where we evaluated the collapsed logo and structure model (Fig. 1, red arrows). For convenience, in the database in the "Family" tab (Fig. 2), the "origin" of the family is recorded, which includes the parameters used and information about any customized steps used in family model construction.

Two large and highly similar Pfam families PF00069 (Pkinase) and PF07714 (PK_Tyr_Ser-Thr) were combined into one family of classical kinases PKLF000033 (Pkinase). Instead of using phmmer, here we employed HMMsearch (with an E-value threshold of 0.0001) and HMM [33] derived from seed alignments (PF00069 and PF07714) from the Pfam database [19]. This HMM was employed to gather homologs, which were then clustered at a 30% sequence identity level.

Each family is assigned a unique identifier (Additional file 1: Table S1; Fig. 2), beginning sequentially with the abbreviation “Protein Kinase-Like—PKL + F” followed by the family's ordinal number. Additionally, each family possesses its own distinctive name.

## Results

### Database implementation

All PKL families and their relevant information were deposited into a local postgreSQL database. The KINtaro database website (http://bioinfo.sggw.edu.pl/kintaro/) was developed with the Django framework on a Linux machine. All KINtaro data is accessible for all users without registration or login. One can register to maintain sequence search history.

### What KINtaro provides

KINtaro offers concise descriptions in family cards (Fig. 2) along with sequence logos collapsed to representative sequences [36] with annotated catalytic residues (when possible) corresponding to canonical kinase catalytic residues. The active site assignments (as originally described by Hanks) is based on literature [1], family sequence logos, 3D structure models, known structures and homology. Family structure models are provided, generated using either AlphaFold2 [31] or ESMfold [32]. Additionally, curated representative protein structures from PDB and individual PKL domain structures are provided [38]. The database also includes, for every family, a HMM sequence model, sets of full and clustered sequences of family members, accompanied by their alignments, full sequences containing the PKL domain and links to external databases. Family HMMs can be used to enrich, for example, genomic annotations. The provided sets of PKL sequences can be used for example, for finding new families (e.g. by cluster analysis through quasi-distances between sequences [39]). Structures and models, as mentioned earlier, can be used to search for distant kinase homologs [30]. Such a well-curated dataset can support research into novel (pseudo)enzymatic PKL families.

### PKL family search in KINtaro

KINtaro enables users to conduct PKL domain searches with their own sequences using HMMscan (HMMER [33]). KINtaro also is searchable by keywords.

## Conclusions

The family of proteins with the PKL fold is continuously expanding. In 2020, we counted over 50 families [40], and in 2022, nearly 70 [29]. Primarily composed of kinases, this group also includes proteins with diverse enzymatic functions and proteins with non-enzymatic roles [15, 16]. To summarize, our database represents a meticulously curated compilation of PKL proteins, serving as a comprehensive and up-to-date resource for information on this rapidly expanding protein superfamily.

## Limitations

For some novel families, the PKL assignment is not experimentally confirmed but only predicted by sequence and structure similarities.

### Supplementary Information

**Additional file 1:** **Table S1.** KINtaro families. Columns: ID_family—KINtaro family id, name_family—KINtaro family name, pfam_id—pfam family id, interpro_id—interpro family id.

## Data Availability

The database and all information can be found at http://bioinfo.sggw.edu.pl/kintaro/. Apart from that, authors are always welcome to share the data required for reviewers and other researchers.

## Abbreviations

PDB: Protein Data Bank
PKL: Protein Kinase-like
pLDDT: Per-residue confidence metric of structure models
